# Effect of Lactate on the Microbial Community and Process Performance of an EBPR System

**DOI:** 10.3389/fmicb.2019.00125

**Published:** 2019-02-18

**Authors:** Francisco J. Rubio-Rincón, Laurens Welles, Carlos M. Lopez-Vazquez, Ben Abbas, Mark C. M. van Loosdrecht, Damir Brdjanovic

**Affiliations:** ^1^Sanitary Engineering Chair Group, Department of Environmental Engineering and Water Technology, UNESCO-IHE Institute for Water Education, Delft, Netherlands; ^2^Department of Biotechnology, Delft University of Technology, Delft, Netherlands

**Keywords:** *Candidatus* Accumulibacter phosphatis, *Tetrasphaera*, poly-phosphate accumulating organism, lactate, glycogen accumulating metabolism

## Abstract

*Candidatus* Accumulibacter phosphatis is in general presented as the dominant organism responsible for the biological removal of phosphorus in activated sludge wastewater treatment plants. Lab-scale enhanced biological phosphorus removal (EBPR) studies, usually use acetate as carbon source. However, the complexity of the carbon sources present in wastewater could allow other potential poly-phosphate accumulating organism (PAOs), such as putative fermentative PAOs (e.g., *Tetrasphaera*), to proliferate in coexistence or competition with *Ca*. Accumulibacter. This research assessed the effects of lactate on microbial selection and process performance of an EBPR lab-scale study. The addition of lactate resulted in the coexistence of *Ca*. Accumulibacter and *Tetrasphaera* in a single EBPR reactor. An increase in anaerobic glycogen consumption from 1.17 to 2.96 C-mol/L and anaerobic PHV formation from 0.44 to 0.87 PHV/PHA C-mol/C-mol corresponded to the increase in the influent lactate concentration. The dominant metabolism shifted from a polyphosphate-accumulating metabolism (PAM) to a glycogen accumulating metabolism (GAM) without EBPR activity. However, despite the GAM, traditional glycogen accumulating organisms (GAOs; *Candidatus* Competibacter phosphatis and *Defluvicoccus*) were not detected. Instead, the 16s RNA amplicon analysis showed that the genera *Tetrasphaera* was the dominant organism, while a quantification based on FISH-biovolume indicated that *Ca*. Accumulibacter remained the dominant organism, indicating certain discrepancies between these microbial analytical methods. Despite the discrepancies between these microbial analytical methods, neither *Ca*. Accumulibacter nor *Tetrasphaera* performed biological phosphorus removal by utilizing lactate as carbon source.

## Introduction

Enhanced biological phosphorus removal (EBPR) or chemical phosphorus removal process or a combination of both processes aims to minimize the proliferation of cyanobacteria and algae in receiving surface water bodies by minimizing the phosphate load to these surface waters (Yeoman et al., 1988). The EBPR process is usually preferred as it offers a more ecological and economical approach (Inc. Metcalf & Eddy et al., 2003).

EBPR is carried out by poly-phosphate accumulating organisms (PAOs) capable of storing phosphorus beyond their growth requirements. PAOs proliferate in activated sludge wastewater treatment plants (WWTP) by recirculating the sludge through anaerobic and anoxic/oxic conditions while directing the influent rich in volatile fatty acids (VFAs) to the anaerobic stage (Barnard, 1975). Previous research has suggested that *Candidatus* Accumulibacter phosphatis (PAOs which anaerobically store VFAs), *Tetrasphaera* (able to ferment different carbon sources) and most recently *Thiothrix caldifontis* (mixotrophic PAOs capable to use sulfide as energy source) can be potential organisms responsible for the biological removal of phosphorus in wastewater treatment plants (Hesselmann et al., 1999; Nguyen et al., 2011; Rubio-Rincón et al., 2017b). Despite the different potential PAOs observed, only the relative bio abundance of *Ca*. Accumulibacter phosphatis has been associated with a good EBPR in WWTP with different configurations located worldwide (Kong et al., 2002; Zilles et al., 2002; Saunders et al., 2003; He et al., 2005; Wong et al., 2005; López-Vázquez et al., 2008).

When acetate or propionate is anaerobically fed, *Ca*. Accumulibacter stores the carbon source as a mixture of poly-β-hydroxyalkanoates (e.g., 3HB, 3HV). The energy needed under anaerobic conditions (*as* ATP) is provided by the hydrolysis of Poly-P and glycogen consumption, while the consumption of glycogen also provides the reducing equivalents (*as* NADH) needed. When an electron acceptor (O_2_, or NO_2_) is present, *Ca*. Accumulibacter produces energy and reducing equivalent through the oxidation of PHA to replenish its Poly-P and glycogen storage pools, for maintenance and growth (Smolders et al., 1995b). Similarly, *Tetrasphaera* is capable to putative ferment distinct carbon sources anaerobically, presumably using either glycogen, amino acids and/or poly-β-hydroxyalkanoates as the main storage compounds (Kristiansen et al., 2013; Marques et al., 2017). While is unlikely that *Tetrasphaera* can store acetate as poly-β-hydroxyalkanoates (PHA) its formation cannot be excluded. As Kristiansen et al. (2013) identified the genes *phaA* and *phaB* in the metagenome of *T. australiensis, T. elongata*, and *T. jenkinsii*, and the genes *phaA, phaB*, and *phaC* in the metagenome of *Tetrasphaera japonica*.

Interestingly, it seems that *Tetrasphaera* is capable to remove phosphorus biologically either under anaerobic or aerobic conditions (Kristiansen et al., 2013; Marques et al., 2017). The aerobic removal of phosphorus observed by *Tetrasphaera* seems to be linked to the anaerobic storage of glycogen and its subsequent use as energy source under aerobic conditions for the replenishment of its Poly-P pools and growth (Kristiansen et al., 2013). On the contrary, when energy is anaerobically provided via the fermentation of carbon sources *Tetrasphaera* accumulate phosphorus anaerobically (Marques et al., 2017). Nevertheless, due to the different metabolic response of *Tetrasphaera* to a single or a mixture of fermentable carbon sources, the actual metabolic use of energy generate via fermentation by *Tetrasphaera* remains unclear (Marques et al., 2017).

Previously, the cultivation of *Ca*. Accumulibacter phosphatis has been achieved mainly with acetate, propionate or a mixture of these VFAs while during short-term other carbon sources such as: glucose, lactate, methanol, ethanol, glycerol, among others have been assessed (Satoh et al., 1994; Smolders et al., 1994; Oehmen et al., 2004; Pijuan et al., 2009). The short-term effects of the carbon sources offers a good indication of the anaerobic metabolic response of *Ca*. Accumulibacter. However, it does not provide the means to assess the potential adaptation or selection of known PAO (and even new PAO as e.g., a new clade of *Accumulibacter* or a new genera as *Tetrasphaera*) to other carbon sources. Hence, there is the need to study the long-term effects of different carbon sources on the metabolism of PAO, which can be present in wastewater and may affect the EBPR process performance. For ethanol, methanol, and glucose these studies are available (Puig et al., 2008; Tayà et al., 2012). However, lactate as a potential important fermentation product form sugars has not been evaluated yet.

Following the idea that certain PAOs can perform differently according to the carbon source fed, previous research focused on the use of glucose as a carbon source. Nevertheless, the results seem inconclusive and contradictory (Jeon and Park, 2000; Wang et al., 2002; Pijuan et al., 2009; Zengin et al., 2010). Wang et al. (2002) reported a stable EBPR with glucose and observed that during the first minutes of anaerobic conditions, glucose is taken up and stored as glycogen (glycogen synthesis). Hereafter glycogen start to be consumed and poly-β-hydroxyalkanoates (PHAs) start to be formed. While some researchers achieved stable biological P removal with glucose as carbon source (Wang et al., 2002), others observed a deterioration of their system caused by a switch from a poly-phosphate accumulating metabolism to a glycogen accumulating metabolism (Zengin et al., 2010). Jeon and Park (2000) previously suggested that this could be caused because PAO stored the lactate generated from the fermentation of glucose (produced by a fermentative group of bacteria). Zengin et al. (2010) did observe that lactate was formed via the fermentation of glucose, but separate batch tests performed with lactate did not result in the biological removal of phosphorus. On the contrary, through short-term batch tests, Satoh et al. (1994) observed that their PAO culture (originally cultivated with acetate) was capable to anaerobically store lactate after a short-term exposure to this compound (largely as PHV) by obtaining the required ATP mainly through the hydrolysis of Poly-P, which was subsequently taken up under aerobic conditions. Thus, it seems that the capability of certain PAOs to accumulate polyphosphate with lactate as carbon source is ambiguously described in literature.

The ability of PAO to anaerobically use lactate and the microbial selection caused by the presence of lactate, is important and needs further study. Lactate can be present in wastewater treatment plants due to the fermentation of other organic compounds such as glucose, or by the discharge of industrial effluents (e.g., dairy industry) (Demirel et al., 2005; Vollertsen et al., 2006). Furthermore, lactate is a simple fermentable carbon substrate (can be fermented to propionate and acetate) which could enhance the growth of putative fermentative PAOs (*Tetrasphaera*) and their possible symbiosis with well-known PAOs (*Ca*. Accumulibacter). Thus, this research aimed to assess the effect of lactate fed together with volatile fatty acids (acetate and propionate) and as sole carbon source, during the operation of a bio reactor aimed to obtain an EBPR process evaluated based on (i) microbial community, (ii) process performance, and (iii) microbial metabolism.

## Materials and Methods

### Operation of the Reactor

Activated sludge from the Harnaschpolder wastewater treatment plant (Den Horn, The Netherlands) was used as inoculum. The biomass was cultivated in a 3.0 L double-jacket Applikon reactor with a working volume of 2.5 L. The bioreactor was operated for over 150 days as a sequencing batch reactor (SBR) in cycles of 6 h, from which 2 h were anaerobic, 2.5 h aerobic and 1.5 h used for settling and effluent withdrawal. At the start of each cycle, 1.25 L of synthetic medium was fed into the reactor. The hydraulic retention time in the bioreactor was of 12 h. Approximately 42 mL of mixed liquor suspended solids (MLSS) were removed per cycle, which resulted in an overall solids retention time (SRT) of 15 d. The pH was adjusted to 7.6 ± 0.1 through the automatic addition of 0.1M HCl and 0.1M NaOH solutions. To suppress the development of GAO (Carvalheira et al., 2014), the dissolved oxygen concentration was kept at around 10% of the saturation concentration via the automatic supply of either compressed air or nitrogen gas. The temperature was controlled at 20 ± 1°C by recirculating water through the double jacket of the reactor. Ortho-phosphate, volatile fatty acids (VFAs), sulfate, sulfide, mix liquor total and volatile suspended solids (MLSS and MLVSS) were measured twice per week at the end of each phase (anaerobic/aerobic). When no changes were observed in the previous parameters for at least 3 SRT, it was assumed that the system had reached pseudo-steady state conditions (Figure S1).

### Synthetic Medium

The synthetic medium fed to the reactor contained per liter 400 mg COD/L, 107 mg NH_4•_Cl (28mg NH_4_-N/L), 112 mg NaH_2_PO_4_•H_2_O (25 mgPO_4_-P/L), 1241 mg MgSO_4_•7H_2_O (498 mg ${\text{SO}}_{4}^{2-}$/L), 14 mg CaCl_2_•2H_2_O (4 mg Ca^+^/L), 36 mg KCl (19 mg K^+^/L), 1 mg yeast extract, 20 mg N-allylthiourea (ATU) and 300 μL of a trace element solution prepared according to Smolders et al. (1994). The carbon source was fed either as a mixture of acetate, propionate, and lactate or solely as lactate according to the corresponding experimental phase of study. During the experimental phase 1, 210 mg COD/L of acetate, 70 mg COD/L of propionate and 120 mg COD/L of lactate were supplied as carbon sources. During the experimental phases 2 and 3, 400 mg COD/L of lactate was fed as carbon source. The different lactate: COD fractions were chosen in order to represent two scenarios on EBPR systems. The lower concentration was used to slowly acclimatize and select a specific PAO that could potentially use lactate for Bio-P removal. Still, acetate and propionate were also added as carbon sources in order to avoid a possible complete and sudden loss of the EBPR activity. On the other hand, the higher concentration of lactate was fed to asses if lactate could be used as sole carbon source for Bio-P removal.

### Experimental Phases

The first experimental phase was carried out once the system reached pseudo steady-state conditions using a mixture of acetate, propionate, and lactate as carbon sources. The second experimental phase, aimed to assess the short-term exposure of the enriched culture to the presence of lactate as carbon source, was conducted in the first cycle that lactate was fed as sole carbon source. Finally, the third experimental phase was carried out once the system reached pseudo steady-state conditions with lactate as the sole carbon source fed into the system. Besides the carbon source fed, the operational conditions in the 3 experimental phases were similar. The process performance of each experimental phase was assessed through a cycle test. During a cycle test, VFAs, lactate, orthophosphate, PHA, Glycogen, sulfate, sulfide, MLSS, and MLVSS were measured at different time intervals during the anaerobic and aerobic phases.

### Stoichiometry and Kinetic Parameters of Interest

The phosphate release per VFA consumption ratio (P-mol/C-mol), PHA stored per VFA consumed (C-mol/C-mol), glycogen consumed per VFA consumed (C-mol/C-mol), fraction of PHV per PHA (PHV/PHA) and fraction of PHB per PHA (PHB/PHA) were calculated based on the observed net conversions between their initial and final concentrations during the anaerobic phase. The observed growth rate and mass balances were calculated as described in Henze et al. (2008) based in all corresponding inflows and outflows during the cycle analysis.

All kinetic rates were calculated by linear regression as described in Smolders et al. (1995a). All rates reported are the maximum observed and checked with the ANOVA test considering 95% confidence interval (Table S1 and Figure S2).

The kinetic rates of interest were:

$q_{COD}^{MAX}$: Maximum specific VFA consumption rate in C-mmol/ gVSS.h;

$q_{PO_{4},AN}^{MAX}$: Maximum specific total phosphate release rate in P-mmol/gVSS.h;

*q*_*P*_*O*__4_, *VFA*_: Maximum specific phosphate release rate for VFA uptake in P-mmol/gVSS.h, estimated as

(1) $$\begin{array}{lll} q_{PO_{4},VFA} & = & q_{PO_{4},AN}^{MAX}{-m}_{PO_{4},AN}; \end{array}$$

*m*_*P*_*O*__4_, *AN*_: Anaerobic specific phosphate release rate due to maintenance purposes (after VFA consumption) in P-mmol/gVSS.h

*q*_*P*_*O*__4_, *Ox*_ : Aerobic phosphate uptake rate in P-mmol/gVSS.h.

### Analyses

All samples for the determination of soluble or dissolved compounds were filtered through 0.45 μm pore size filters. PHA and glycogen were measured as described in Rubio-Rincón et al. (2017b). Orthophosphate, sulfide and ammonia were measured according to the standard methods for the examination of wastewater (APHA, 2005). Sulfate was measured in an Ion Chromatography system equipped with a Dionex Ionpack AS4A-SC column (Dreieich, Germany). Acetate (HAc) and propionate (HPr) were measured in a gas chromatography system G420-C (Nieuwegein, The Netherlands). Lactate was measured in a high-performance liquid chromatography (HPLC) using a Trace 2000 chromatograph (Thermo Electron S.P.A., Milan, Italy).

### Microbial Identification

#### Analyses of Bacterial Community Compositions by V4-V6 16S rRNA Gene-Based Amplicon Sequencing

Genomic DNA (gDNA) was extracted using the Ultraclean Microbial DNA extraction kit supplied by MOBIO laboratories Inc. (CA, USA) according to the manufacturer's protocol except that the bead-beating was substituted by a combination of 5 min heating at 65°C and 5 min beat-beating to ensure maximum yields. To check for quality and quantity, the gDNA extracts were loaded onto a 1% agarose gel in 1x TAE running buffer. Analysis of the extracted gDNA showed a large high molecular weight fraction and well-visible DNA yields in comparison to the Smart ladder (Eurogentech Nederland b.v.).

The extracted gDNA was subsequently used for a two-step PCR reaction targeting the 16S rRNA gene of most bacteria and archaea, using the primers U515F (5′-GTGYCAGCMGCCGCGGTA-3′) and U1071R (5′-GARCTGRCGRCRRCCATGCA-3′) following Wang and Qian (2009). The first amplification step was performed to enrich for 16S rRNA genes, via quantitative PCR (qPCR). The qPCR reaction comprised 2x iQ™ SYBR® Green Supermix (Bio-rad, CA, USA), 500 nmol L^−1^ primers each, and 1–50 ng gDNA template added per well (final volume of 20 μL by adding MiliQ water). The qPCR program went along a first denaturation at 95°C for 5 min followed by 20 cycles of denaturation at 95°C for 30 s, annealing at 50°C for 40 s and elongation at 72°C for 40 s, prior to final elongation at 72°C for 7 min. During the second step, 454-adapters (Roche) and MID tags at the U515F primer, were added to the products of step one. This protocol was similar to the ones previously described, but only Taq PCR Master Mix (Qiagen Inc, CA, USA) was used. The program was run for 15 cycles, the template, product from step one was used as template DNA and diluted ten times. After the second amplification, 12 PCR products were pooled in equimolar ratio and purified over an agarose gel using a GeneJET Gel Extraction Kit (Thermo Fisher Scientific, The Netherlands). The resulting library was send for 454 sequencing and run in 1/8 lane with titanium chemistry by Macrogen Inc. (Seoul, Korea).

After sequencing, the reads library was imported into the CLC genomics workbench v7.5.1 (CLC Bio, Aarhus, DK) and (quality, limit = 0.05 and max. two ambiguities allowed) trimmed to a minimum of 200 bp and average of 284 bp. After trimming, the datasets were de-multiplexed resulting in 12 samples with an average of 7,800 reads per sample. A build-it SILVA 123.1 SSURef Nr99 taxonomic database was used for BLASTn analysis on the reads under default conditions. To identify chimeric sequences we used the online tool DECIPHER (Wright et al., 2012). Sequences were only included if the *E*-value was sufficient low (<E-^50^). The top result was imported into an excel spreadsheet and used to determine taxonomic affiliation and species abundance.

#### Molecular Analysis of PAO Clades by PCR on the *ppk1* Functional Gene

A direct PCR was performed to identify the “Ca. Accumulibacter” clade enriched in the biosystem based on the polyphosphate kinase (*ppk1*) functional gene as described by McMahon et al. (2007). The PCR amplicons were produced using ACCppk1-254F (5′-TCAC CACC GACG GCAA GAC-3′) and ACCppk1-1376R (5′-TCGA TCAT CAGC ATCT TGGC-3′) primers, and (Sanger) sequenced by BaseClear, Leiden, the Netherlands. Both strands were quality checked and found non-ambiguous. Subsequently both were aligned to yield a high quality, near complete, ppk1 gene. The phylogenetic tree was constructed using the neighbor joining method implemented in the CLC genomics workbench package, as described by Saad et al. (2016). In total 332 amino-acid positions were used for calculations.

#### Fluorescence *in situ* Hybridization (FISH)

In order to visually corroborate the relative bio abundance of the most relevant microbial communities at the different experimental phases, fluorescence *in situ* hybridization (FISH) analyses were performed as described by Amann (1995). In order to target all bacteria, equal amounts of EUB 338, EUB338 II and EUB 338 III probes were mixed (EUB MIX) and applied (Nielsen et al., 2009). Because of the relative low fraction of *Propionivibrio* observed with 16S rRNA gene-based amplicon sequencing (~5% only observed during the exp. phase 1), and also because the PAO 651 FISH probe suggested by Albertsen et al. (2016) only targets 71% of the species of the genus *Ca*. Accumulibacter, the mix probe of PAO 651, PAO 462 and PAO 846 developed by Crocetti et al. (2000) was used to target the *Ca*. Accumulibacter genus (with a 89% target). *Candidatus* competibacter a well-known GAO observed in EBPR systems was targeted with the GB probe developed by Kong et al. (2002). *Defluvicoccus* clusters 1 and 2 were identified with the TFO-DF215, TFO-DF618, DF988 and DF1020 probes (Wong et al., 2004; Meyer et al., 2006). Vectashield containing a DAPI concentration was used to preserve the fluorescent signal and stain all organisms present (Nielsen et al., 2009). FISH quantification of each probe was performed by image analysis of 20 random pictures taken with an Olympus BX5i microscope and analyzed with the software Cell Dimensions 1.5. The standard error of the arithmetic mean was calculated based on the standard deviation divided by the square root of the number of pictures taken.

## Results

### Anaerobic and Aerobic Stoichiometry and Kinetics

The EBPR was inoculated with activated sludge from the Harnaschpolder EBPR wastewater treatment plant (Den Horn, The Netherlands). After 34 days of operation, the system reach a stable phosphorus release of 150 ± 3.2 mg P/L and MLVSS of 3,027 ± 234 mg SS/L (Figure S1). At day 83 (~3 SRT after pseudo steady state) a cycle was analyzed (experimental phase 1). Right after the carbon source was change to solely lactate and a cycle was analyzed (experimental phase 2) to assess the shock response of the bacteria to the change of carbon source. After 3SRT of lactate feeding, at day 150 the last cycle was analyzed (experimental phase 3) to assess the acclimatization/selection of microbial community to the new substrate.

Table 1 (and Table S1 with statistical analysis) summarizes the specific rates and stoichiometric parameters of interest observed during the three experimental phases. In the first experimental phase (when the carbon source was composed of acetate, propionate and lactate in a COD-ratio of 3.0:1.0:1.7, respectively), the influent 6.25 C-mmol/L was taken up in the anaerobic phase at a rate of 3.06 C-mmol/gVSS.h. The carbon source was stored as PHB and PHV (3.12 C-mml/L and 2.52 C-mmol/L, respectively). Simultaneously, 4.40 P-mmol/L of Poly-P were hydrolysed at a rate of 2.82 P-mmol/gVSS.h (*q*_*P*_*O*__4_, *VFA*_) to provide most of the energy required for carbon uptake and 0.05 P-mmol/gVSS.h (*m*_*P*_*O*__4_, *AN*_) were released to cover the corresponding anaerobic maintenance requirements. Approximately 1.17 C-mmol/L of glycogen was consumed presumably as a source of energy and reducing equivalents. When the system reached pseudo steady-state conditions, the observed growth was 0.28 gVSS/gCOD and the anaerobic COD balance closed up to 95%. In the aerobic period, 4.58 P-mmol/L of phosphate was taken up at a rate of 1.06 P-mmol/gVSS.h.

**Table 1:** Anaerobic and aerobic biomass specific rates and stoichiometry observed with acetate, propionate, and lactate was fed as carbon sources (Exp. Phase 1), in the first cycle (Exp. Phase 2) and after reaching pseudo steady-state conditions (Exp. Phase 3) with lactate as the sole carbon source in EBPR enrichments in anaerobic-aerobic SBR process.

	**Experimental phase**	**Units**	**1 Mixture of Ac:Pr:Lac**	**2 Lactate 1st cycle**	**3 Lactate**
	**Parameter**				
Biomass	MLVSS	mg/L	2,780	2,757	3,257
	MLSS	mg/L	4,633	4,503	3,983
	Y_obs_	gVSS/gCOD	0.28	N.A.	0.33
Net anaerobic conversions	COD_uptake_	C-mmol/L	6.25	3.93	6.25
	PHB	C-mmol/L	3.12	0.62	0.62
	PHV	C-mmol/L	2.52	2.43	4.11
	PHA	C-mmol/L	5.64	3.06	4.72
	Gly	C-mmol/L	1.17	0.24	2.96
	P_rel_	P-mmol/L	4.40	5.14	1.09
	COD_balance_	%	95%	NA	87%
Anaerobic ratios	PHV/PHA	C-mol/C-mol	0.44	0.79	0.87
	P/VFA	P-mol/C-mol	0.70	1.30	0.11
	Gly/VFA	C-mol/C-mol	0.18	0.06	0.47
Rates	*q*_*COD*_	C-mmol/gVSS.h	3.06	0.96	2.71
	$q_{PO4,AN}^{MAX}$	P-mmol/gVSS.h	2.87	1.34	0.33
	*m*_*PO*4, *AN*_	P-mmol/gVSS.h	0.05	NA	0.05
	*q*_*PO*4, *VFA*_	P-mmol/gVSS.h	2.82	NA	0.28
	*q*_*PO*4, *Ox*_	P-mmol/gVSS.h	1.06	0.25	0.21

During the first cycle when lactate was fed as sole carbon source (Exp. Phase 2), only 3.93 C-mmol/L out of 6.25 C-mmol/L fed were taken up at a rate of 0.96 C-mmol/gVSS.h. Neither acetate nor propionate accumulation was observed. About 2.43 C-mmol/L of PHV and 0.62 C-mmol/L of PHB formation were measured in the biomass. A lower amount of glycogen was consumed (0.24 C-mmol/L) compared to Phase 1. In contrast, 5.14 P-mmol/L of Poly-P were hydrolysed at a maximum specific rate of 1.34 P-mmol/gVSS.h ($q_{PO_{4},AN}^{MAX}$). However, in the aerobic stage, only 1.96 P-mmol/L were taken up at rate of only 0.25 P-mmol/gVSS.h.

**Figure 1:**
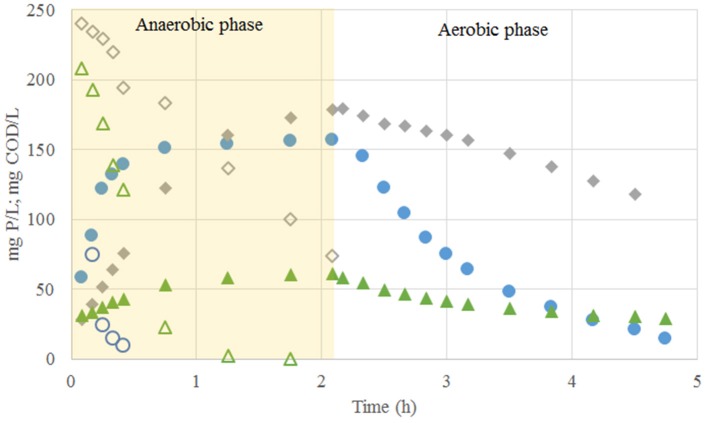
Profiles of carbon (open markers) and phosphate concentrations (closed markers) observed in the experimental phase No. 1 (blue open and closed circles), phase No. 2 (gray open and closed diamonds) and phase No. 3 (green open and closed triangles).

Once the system reached pseudo steady-state conditions (Exp. Phase 3), the 6.25 C-mmol/L fed were consumed anaerobically at a rate of 2.71 C-mmol/gVSS.h and, like in the previous experimental phases, neither acetate nor propionate accumulation was observed. Out of the 6.25 C-mmol/L taken up, about 4.11 C-mmol/L was stored as PHV and only 0.62 C-mmol/L as PHB. 1.09 P-mmol/L of Poly-P was hydrolysed at a rate of 0.28 P-mmol/gVSS.h when the carbon source was taken up (*q*_*P*_*O*__4_, *VFA*_). Interestingly, the glycogen consumption increased to 2.96 C-mmol/L. The COD balance closed up to 87%, and the observed growth was 0.33 gVSS/gCOD. During the aerobic conditions, an incomplete phosphorus uptake of up to 1.03 P-mmol/L at rate of 0.21 P-mmol/gVSS.h was observed (Figure 1). In none of the experimental phases an increase on the suspended solids in the effluent was detected.

### Effect of Lactate on Microbial Community Dynamics

**Figure 2:**
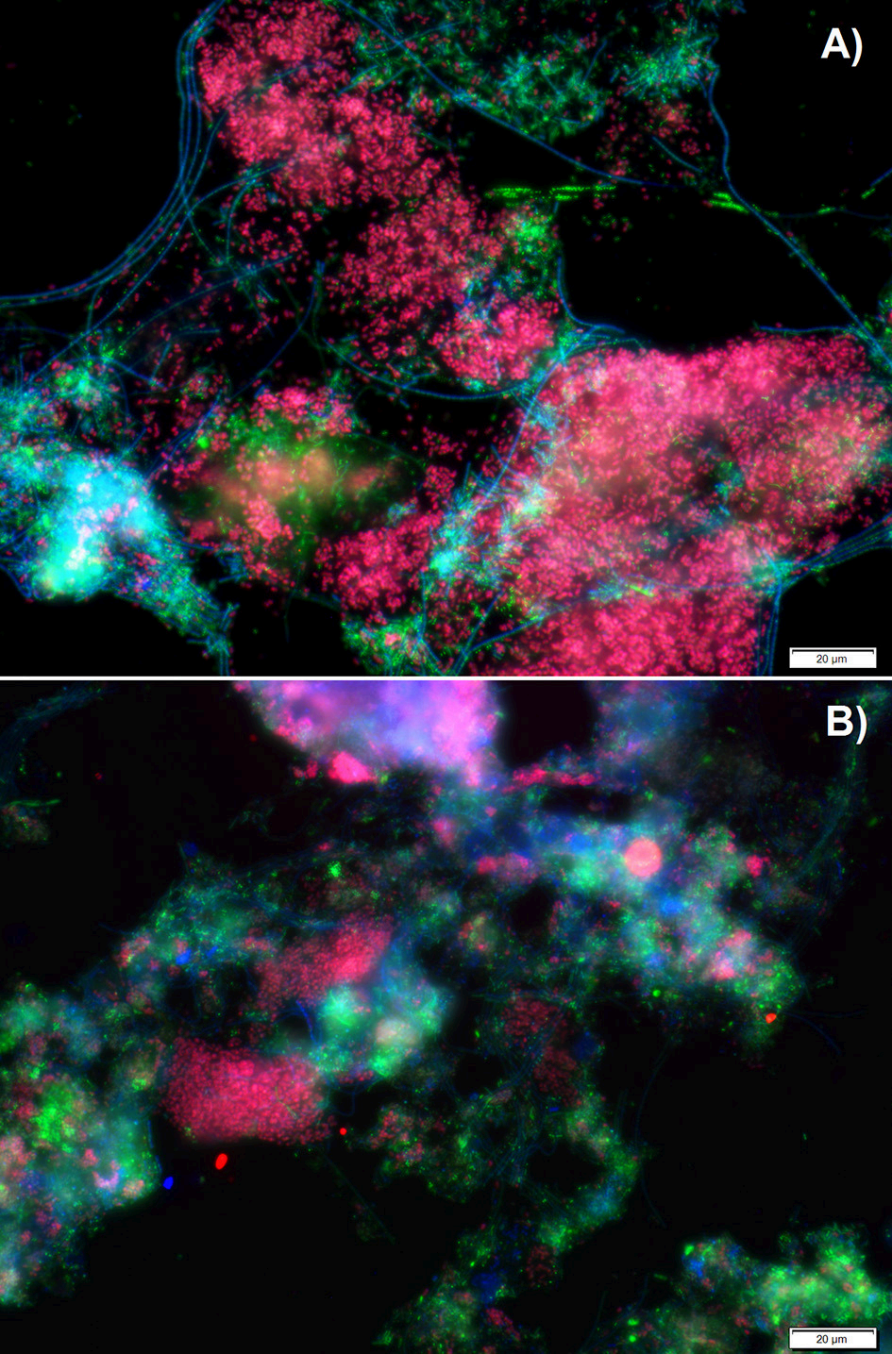
Microbial characterization via fluorescence *in situ* hybridization (FISH) analyses when a mixture of acetate, propionate and lactate was fed **(A)** or only lactate **(B)** as carbon source. In green all organisms (DAPI), in blue (Cy5) most bacteria (EUB I, II, and III), in red (Cy3) *Ca*. Accumulibacter (PAO 651,846 and 462), and in yellow (Fluos) GAOs (GB, TFO-DF215, TFO-DF618, DF988 and DF1020).

**Figure 3:**
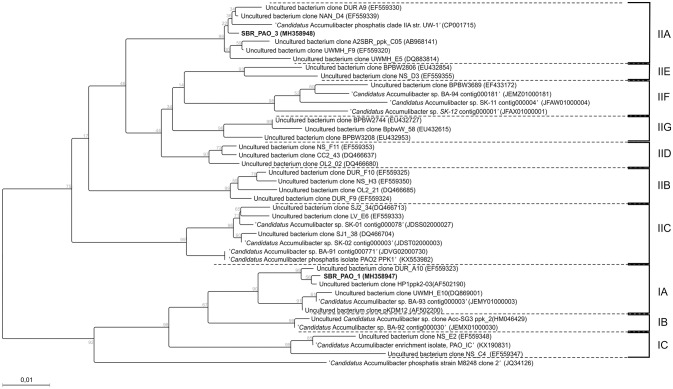
Phylogenetic tree of genus *Ca*. Accumulibacter based on the ppk gene. Samples SBR_PAO_1 and SBR_PAO_3, were taken when the system reached pseudo steady-state conditions when acetate, propionate and lactate or only lactate was fed anaerobically as carbon source, respectively.

Fluorescence *in situ* hybridization (FISH) analyses were performed to assess the relative bio abundance of *Ca*. Accumulibacter phosphatis, assumed to be one of main PAOs responsible for the biological removal of phosphorus in full-scale activated sludge systems. Figure 2 shows a representative image of the microbial composition when a mixture of acetate, propionate and lactate (experimental phase 1) or only lactate (experimental phase 3) was fed as carbon source. The relative bio abundance of *Ca*. Accumulibacter phosphatis (targeted with the PAO 651, 846 and 462 probes) with regard to the whole microbial populations (targeted by DAPI) decreased from 79 ± 3 to 61 ± 3% from the experimental phase 1 to 3. GAO (*Ca*. competibacter and *Defluvicoccus*) were barely observed in any of the experimental phases (with an estimated abundance of <1% according to the GAO mix probe used). Further *Ca*. Accumulibacter identification based on the *ppk1* functional gene showed that the main subclade switched from subclade IA (SBR_PAO_1) to subclade IIA (SBR_PAO_3) when the carbon source changed from a mix of acetate, propionate and lactate to only lactate (Figure 3).

To support the previous observations, the results gathered from the 16S rRNA gene-based amplicon sequencing tests showed a decrease in the relative abundance of the family *Rhodocyclaceae* (to which *Ca*. Accumulibacter belongs) from 20 to 2% when the carbon source changed to only lactate. Moreover, *Ca*. Accumulibacter could not be detected (<1%) when lactate was fed as the only carbon source (Table 2). On the contrary, the family *Intrasporangiaceae* (to which *Tetrasphaera* belongs) increased their relative abundance from 4 to 27% when solely lactate was fed, so did *Tetrasphaera* that increased from 4 to 16% (from Exp. phase 1 to 3, respectively). The increase in the relative abundance of *Rhodobacteraceae* and *Chitinophagaceae* from Phase 1 to 3 was also significant (from 5 to 17% and 3 to 18%, respectively).

**Table 2:** Read abundance observed at family and genus level by 16S rRNA gene-based amplicon sequencing, during the experimental phase 1 (mixture of COD fed) and phase 3 (only lactate fed as COD).

	**Read abundance**	
	**Phase 1 (%)**	**Phase 3 (%)**
**FAMILY**		
*Flavobacteriaceae*	39	11
*Rhodocyclaceae*	20	2
*Xanthomonadaceae*	10	10
*Sphingomonadaceae*	6	6
*Hydrogenophilaceae*	6	1
*Rhodobacteraceae*	5	17
*Intrasporangiaceae*	4	27
*Erysipelotrichaceae*	3	0
*Chitinophagaceae*	3	18
*Xanthomonadales Incertae Sedis*	2	1
*Comamonadaceae*	2	4
*Anaerolineaceae*	1	4
**GENUS**		
*Flavobacterium*	26	2
*Candidatus* Accumulibacter	13	1
*uncultured*	13	45
*Chryseobacterium*	12	5
*Thermomonas*	7	0
*Sphingopyxis*	7	3
*Thiobacillus*	6	0
*Propionivibrio*	5	0
*Tetrasphaera*	4	16
*Erysipelothrix*	4	0
*Pseudoxanthomonas*	3	2
*Dechloromonas*	2	0

From the family C*hitinophagaceae* the main genera increase was *Niabella* (from 95 to 577 counts, on Exp. phase 1 to 3, respectively), while from the family *Rhodobacteraceae* the genera *Rhodovulum* was detected at experimental phase 3 with 56 counts. None of these genera has been suggested as putative PAO (Stokholm-Bjerregaard et al., 2017), so the role or relationship of *Rhodobacteraceae* and C*hitinophagaceae* with any potential EBPR activity is unclear. On the other hand, Hülsen et al. (2014, 2016) implicated *Rhodobacter* (from the family *Rhodobacteraceae*) as possible organism for the removal of phosphorus. Despite that *Rhodobacter* was not target with FISH, the read abundance by 16SrRNA was only of 6 counts out of 6,723 (Exp. phase 3), which make it unlikely the active presence of this organism in our system. Furthermore, the cultivation conditions of *Rhodobacter* in the studies of Hülsen et al. (2014, 2016) were strongly different from standard EBPR conditions and as many bacteria can store PHA and Poly-P without being an effective EBPR organisms we do not yet consider *Rhodobacter* as a putative EBPR organism.

## Discussion

### Adaptation/Selection of the Microbial Community

According to the fluorescence *in situ* hybridization (FISH) analyses, *Ca*. Accumulibacter remained the dominant microorganisms when the carbon source changed from a mixture of lactate and VFA to only lactate (comprising ~60% of the whole microbial community, on a bio-volume basis). *ppk* analyses showed that the dominant *Ca*. Accumulibacter subclades switched from IA to IIA. Previously, Acevedo et al. (2012) and Welles et al. (2015, 2017) showed that both clades of *Ca*. Accumulibacter could solely rely on glycogen for the generation of ATP and NADH under phosphate limiting conditions. Thus, it seems that *Ca*. Accumulibacter can switch from main metabolic pathway (either GAM or PAM phenotype) according to the environmental conditions of the system. In our study such switch in the metabolic pathway was triggered by the change of carbon source from a mixture of acetate, propionate and lactate to only lactate. Which to be the best of our knowledge has not previously been reported.

Despite the differences in absolute numbers, both the FISH analysis and the 16S rRNA gene-based amplicon sequencing analysis showed a decrease in the abundance *Ca*. Accumulibacter. The differences in the absolute numbers can be explained by biases in the different methods as previously discussed by Valverde-Pérez et al. (2016) and Albertsen et al. (2016). This clearly shows that sequencing data should always be validated with FISH or another molecular methods.

Interestingly, the 16S rRNA gene-based amplicon sequencing analyses showed a shift in the dominant organism from *Ca*. Accumulibacter to *Tetrasphaera* (from the experimental phase No. 1 to No. 3). *Tetrasphaera* has been previously suggested to have a similar EBPR metabolism like *Ca*. Accumulibacter (with feast and famine regimes) (Nguyen et al., 2011) and some species such as *T. japonica* encode the necessary genes (*phaA, phaB*, and *phaC*) for the formation and storage of PHA (Kristiansen et al., 2013). This could explain the higher abundance of *Tetrasphera* observed in this study. Until now however no physiological studies have shown unambiguously Tetrasphaera to store PHA based on acetate uptake. In our study, despite the relative high abundance of *Tetrasphaera* and *Ca*. Accumulibacter in the community, there was no effective phosphate removal observed when lactate was the sole carbon source. A carefully assessment should be done about the ability of fermentative bacteria to accumulate phosphorus beyond growth requirements. One of the main reasons for the accumulation of phosphorus by EBPR bacteria as polymer is to provide energy to the bacteria in the absence of an electron acceptor (Smolders et al., 1994). However the energy requirements for putative fermentative PAOs could be provided by the fermentation itself (Thauer et al., 1977; Marques et al., 2017). Nguyen et al. (2011) suggested that *Tetrasphera* may be a fermentative PAO, based on the 30% abundance of these organisms in WWTP performing EBPR, but without providing effective activity measurements. However, the 30% abundance was quantified by adding up the different biomass fractions estimated with the application of 10 different oligonucleotide probes (with each probe detecting <6.8 ± 2.0% of *Tetrasphera* with regard to EUB) using different formamide concentrations (Nguyen et al., 2011). Due to the high relative percentage reported of the normalize standard deviation (about 30% of the main value) this process may not be optimal to estimate the abundance of the *Tetrasphaera* population. In addition, as observed by Valverde-Pérez et al. (2016) and in this study, there seems to be a strong discrepancy between the estimations carried out with DNA and FISH based quantitative methods, suggesting that conclusions drawn from molecular methods should be accompanied by microbial activity measurements.

### Deterioration of EBPR Systems

In this study, the addition of lactate as a sole carbon source led to the deterioration of the EBPR process performance (Figure 1). In line with our results, Baetens et al. (2001) reported the deterioration of an EBPR system when the carbon source changed from acetate to a mixture of acetate and lactate. They suggest that the deterioration was partially caused by the wash-out of the biomass through the effluent due to the proliferation of filamentous bacteria. However, in this research, an increase in the concentration of suspended solids in the effluent was not observed and no proliferation of filamentous bacteria was observed.

Contrary to these results, the system operated by Baetens et al. (2001) reached full P-removal once the growth of filamentous bacteria was suppressed. The anaerobic P-release/VFA-uptake ratio was 0.21 P-mol/C-mol when feeding the mixture of VFA and lactate, which is lower than the P-release/VFA-uptake ratio of 0.75 P-mol/C-mol observed with only acetate. The lower anaerobic P-release/VFA-uptake ratio suggests the occurrence of another main organism (besides a PAO) or a metabolic switch from a polyphosphate-accumulating metabolism (PAM) to a glycogen-accumulating metabolism (GAM). Unfortunately, due to the lack of glycogen measurements or the identification of the microbial communities in the studies of Baetens et al. (2001) none of the previous hypotheses can be assessed.

Jeon and Park (2000) observed a similar P/VFA ratio like the ones observed in this study and by Baetens et al. (2001) (of 0.12, 0.11, and 0.21 P-mol/C-mol, respectively). Jeon and Park (2000) suggested that the observed EBPR activity was a reflection of the presence of at least two communities, one capable of fermenting glucose and another of storing the fermented products (e.g., acetate, propionate, lactate) at the expenses of poly-P hydrolysis. Likewise, Zengin et al. (2010) observed a switch from a PAM to a GAM within 1 SRT when glucose was fed as a carbon source, indicating a phenotypic change. Despite that Zengin et al. (2010) observed lactate accumulation from the fermentation process (like Jeon and Park, 2000), lactate was not anaerobically consumed nor was phosphorus removed. Contrary to the observations of Zengin et al. (2010) and Wang et al. (2002) reported a stable EBPR process when feeding 1,000 mg/L of glucose with a practically negligible phosphate release per carbon consumed ratio (0.038 up to 0.043 P-mol/C-mol). These observations strongly suggest that the main carbon source stored did not require additional ATP from the hydrolysis of Poly-P. According to the anaerobic stoichiometry reported by Smolders et al. (1994) of 0.50 P-mol released/C-mol of carbon consumed, only 8–9% of the COD fed to the system of Wang et al. (2002) might have been possibly fermented to acetate and subsequently stored to reach the anaerobic ratio of 0.038–0.043 P-mol/C-mol. The ambiguous EBPR activities reported in literature with lactate and glucose as carbon sources suggest that they are strongly dependent on the presence of fermentable organisms capable to produce acetate or propionate.

Yamamoto-Ikemoto and Matsui (1996) observed that sulfate reducing bacteria (SRB) produce acetate via partial oxidation, which generates more energy per mol of sulfur compared to the complete degradation to carbon dioxide (Thauer et al., 1977). Yamamoto-Ikemoto and Matsui (1996) suggested that the acetate produced was used for the biological removal of phosphorus. Similarly, Baetens et al. (2001) observed that sulfate was reduced when a mixture of acetate and lactate was fed. Thus, possibly like in the study of Yamamoto-Ikemoto and Matsui (1996), SRB may provide the extra acetate needed for EBPR processes (Baetens et al., 2001; Rubio-Rincón et al., 2017a). However, in this study neither sulfate reduction nor acetate formation was observed. The absence of a fermentative or acetate producing bacteria could possibly explain the deterioration of the biological removal of phosphorus in this study when lactate was fed as the sole carbon source. Moreover, the presence of a reduced form of sulfur, such as sulfide, in the studies of Baetens et al. (2001) and Yamamoto-Ikemoto and Matsui (1996), could have triggered the growth of *T. caldifontis*, a proposed new mixotrophic PAO, increasing the net phosphorus removal of their systems(Rubio-Rincón et al., 2017b).

### Metabolic Shifts

**Figure 4:**
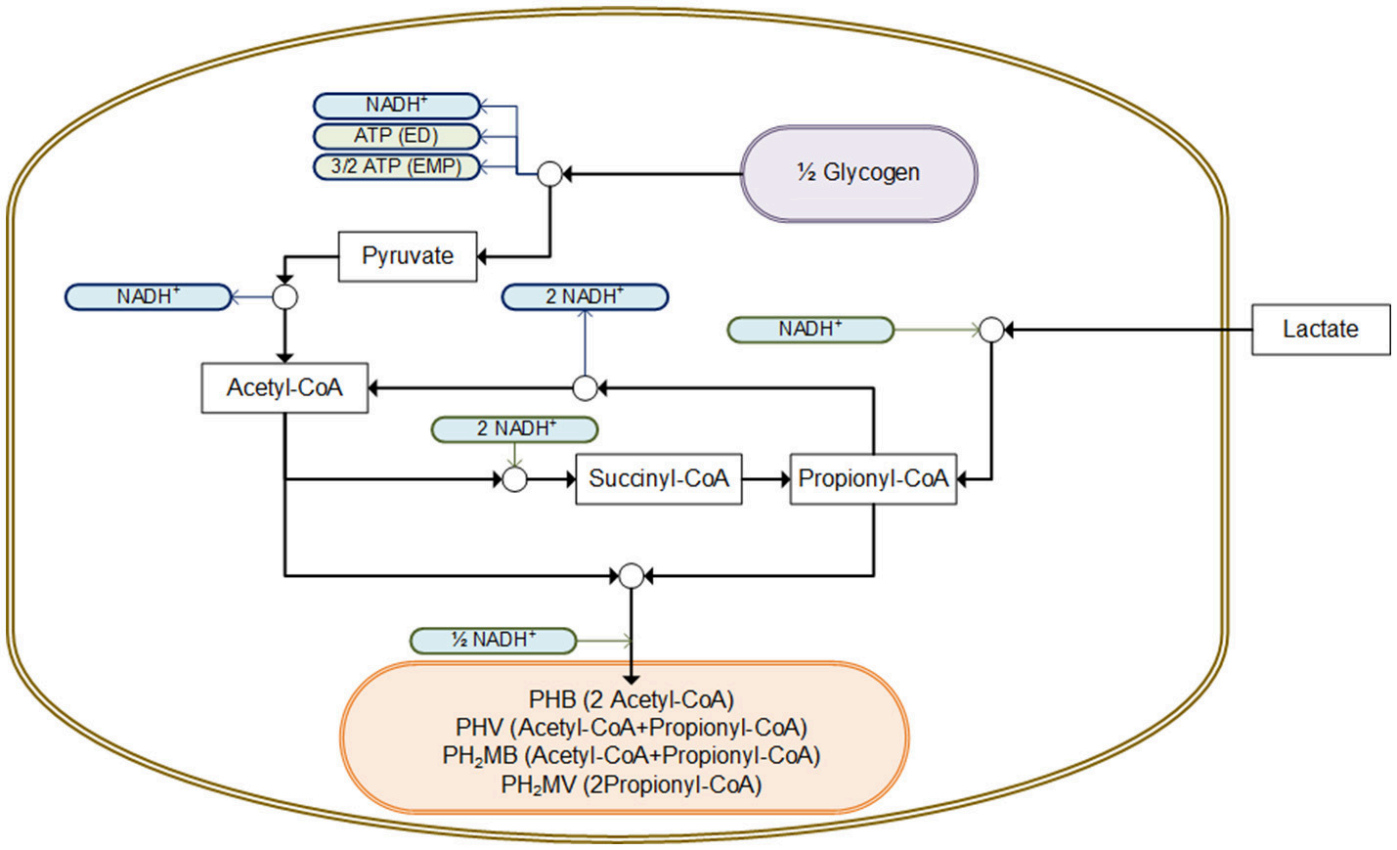
Potential pathway for the intracellular storage of lactate as PHA using glycogen as source of reducing equivalents, adapted from Mino et al. (1998) and Satoh et al. (1992). All compounds presented are consider in C-mol units.

In this research, an increase in the fraction of PHV/PHA was observed when lactate was fed compared to the addition of a mixture of carbon sources (of 0.87 and 0.44 PHV/PHA, respectively). Satoh et al. (1992) also reported an increase in the storage of PHV when lactate was the carbon source. They suggested that PHV was formed via acetyl-CoA and propionyl-CoA through the propionate fermentation pathway (Figure 4). Thus, according to this proposed pathway, the storage of 0.82 mmol PHV (4.11 C-mmol) and 0.15 mmol PHB (0.62 C-mmol) would require ~1.30 mmol of NADH, which in this study could have been provided by the consumption of 0.49 mmol Glycogen (2.96 C-mmol) as it yields 1.48 mmol of NADH.

In the anaerobic metabolism of *Ca*. Accumulibacter with acetate as carbon source, ATP (as a function of the pH) is used for the transport of acetate across the cell membrane and during the conversion of acetate to acetyl-CoA (Smolders et al., 1994). On the contrary, the transport and conversion of lactate to pyruvate does not require ATP (Satoh et al., 1992). On the contrary during standard conditions the oxidation of lactate into pyruvate yield −24 KJ/mol (Metzler, 2003). In addition, the anaerobic consumption of glycogen could provide extra ATP, which yields between −52.5 and −78.7 KJ/mol via the ED or EMP pathway, respectively (Smolders et al., 1994; Metzler, 2003). Overall, from a metabolic point of view, the aforementioned mechanisms suggest that the hydrolysis of poly-P to generate energy, does not seem to be necessary to store lactate as PHA.

Independently of the dominant putative PAO organism present in the experimental phase No. 3 (either *Tetrasphaera* as suggested by 16S rRNA or *Ca*. Accumulibacter as suggested by FISH), they did not contribute to the EBPR process. Recent studies have pointed out that different carbon sources (or mixtures of including glucose, glycine, aspartate, and glutamate) can trigger different metabolic behaviors in *Tetrasphaera* (such as anaerobic phosphorus uptake and anaerobic glycogen replenishment) (Marques et al., 2017). Also, Kristiansen et al. (2013) reported that *Tetrasphaera* was capable of generating energy from the fermentation of glucose and hydrolysis of Poly-P with the anaerobic formation of glycogen and without intracellular PHA accumulation, using the previously formed glycogen to provide energy for Poly-P replenishment under aerobic conditions. However, as observed in this study and elsewhere (Satoh et al., 1992; Mino et al., 1989), the energy produced by the hydrolysis of Poly-P (*as* ATP) is not strictly needed for the storage of lactate as PHA, which could be stored either by *Ca*. Accumulibacter or *T. japonica* (Smolders et al., 1994; Kristiansen et al., 2013). Thus, this study suggests that neither *Ca*. Accumulibacter nor *Tetrasphaera* can perform the biological phosphorus removal when lactate is fed as sole carbon source and following the aforementioned microbial metabolism. Thus, if lactate is the sole carbon source it should be fermented to volatile fatty acids or other suitable compounds that can trigger the EBPR activity.

## Nucleotide Sequence Accession Numbers

The ppk1 gene sequences obtained in this study have been deposited in the GenBank database under accession numbers MH358947 and MH358948. The 16S-rRNA gene amplicon libraries have been deposited as a SRA archive under project PRJNA454812.

## Author Contributions

All authors contributed to the design of the experiments. FR-R performed the experiments, conducted the FISH analysis and drafted the manuscript. BA conducted the 16S rRNA and ppk analysis. All authors critically read and contributed to the final version of the manuscript. All authors read and approved the final manuscript.

### Conflict of Interest Statement

The authors declare that the research was conducted in the absence of any commercial or financial relationships that could be construed as a potential conflict of interest.
