# Supplementary material for: Effect of Lactate on the Microbial Community and Process Performance of an EBPR System
Source: Front Microbiol. 2019 Feb 18;10:125. doi: 10.3389/fmicb.2019.00125 (PMC6387944; doi:10.3389/fmicb.2019.00125)
Supplement: Supplementary file 1 [file Data_Sheet_1.docx]

# Supplementary information

# Effect of Lactate on the microbial community and process performance of an EBPR system

**Rubio-Rincón F.J.^a,b,*^(** 0000-0002-3622-3121)**, Welles L^a^, Lopez-Vazquez C.M.^a^ (**0000-0002-4250-347X)**, Abbas B.^b^ , van Loosdrecht M.C.M.^b^(**0000-0003-0658-4775)**, Brdjanovic D.^a, b^(**0000-0002-5044-3913)

**^a^** Sanitary Engineering Chair Group. Department of Environmental Engineering and Water Technology, UNESCO-IHE Institute for Water Education, Westvest 7, 2611AX Delft, The Netherlands. (E-mail: [f.rubiorincon@un-ihe.org](mailto:f.rubiorincon@unesco-ihe.org); [c.lopezvazquez@un-ihe.org](mailto:c.lopezvazquez@un-ihe.org); laurenswelles@gmail.com; b.brdjanovic@un-ihe.org).

**^b^**. Department of Biotechnology, Delft University of Technology, Van der Maasweg 9, 2629 HZ Delft

(E-mail: [F.J.RubioRincon@tudelft.nl](mailto:F.J.RubioRincon@tudelft.nl); [m.c.m.vanloosdrecht@tudelft.nl](mailto:m.c.m.vanloosdrecht@tudelft.nl); [d.brdanovic@tudelft.nl](mailto:d.brdanovic@tudelft.nl) ).

* Corresponding author: [f.rubiorincon@un-ihe.org](mailto:f.rubiorincon@unesco-ihe.org); [F.J.RubioRincon@tudelft.nl](mailto:F.J.RubioRincon@tudelft.nl)

## Figure S1


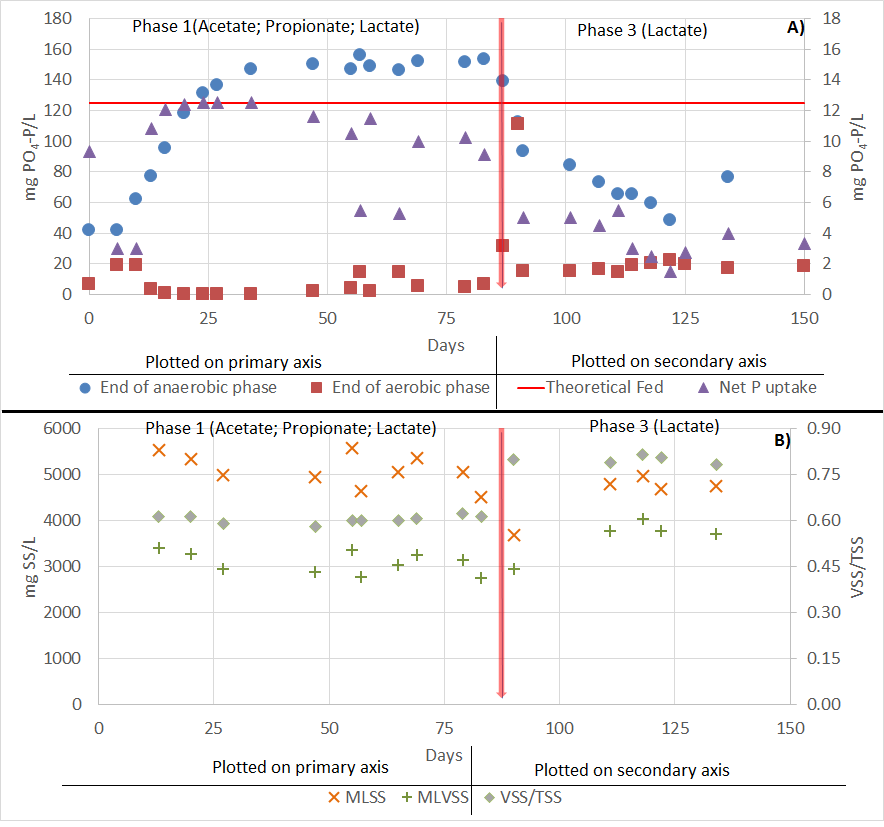


Figure A1.- Monitoring of the bioreactor performance during the 150 days of operation.

## Table S1

Table A1.- Anaerobic and aerobic kinetics rates with ANOVA statistical analysis at 95 % confidence interval and biomass with standard deviation (three samples) in the bioreactor observed when acetate, propionate and lactate was fed as carbon sources (Exp. Phase 1), in the first cycle (Exp. Phase 2) and after reaching pseudo steady-state conditions (Exp. Phase 3) when lactate was fed as the sole carbon source.

|  | **Experimental phase** | **Units** | **1**  **Mixture of**  **Ac:Pr:Lac** | **2**  **Lactate**  **1^st^ cycle** | **3**  **Lactate** |
| --- | --- | --- | --- | --- | --- |
|  | **Parameter** |  |  |  |  |
| **Biomass** | MLVSS | mg/L | 2780±30 | 2757±6 | 3257±85 |
|  | MLSS | mg/L | 4633±85 | 4503±28 | 3983±75 |
| **Rates** | $q_{COD}$ | C-mmol/gVSS.h | 3.06 (R^2^=0.77; F1,2=20;P<0.100) (R^2^=0.99; F1,4=227;P<0.001) | 0.96 (R^2^=0.98; F1,7=236;P<0.001) | 2.71 (R^2^=0.99; F1,4=227;P<0.001) |
|  | $q_{PO4,AN}^{MAX}$ | P-mmol/gVSS.h | 2.87 (R^2^=0.71; F1,4=29;P<0.010) | 1.34 (R^2^=0.92; F1,7=16;P<0.010) | 0.33 (R^2^=0.99; F1,4=27;P<0.001) |
|  | $m_{PO4,AN}$ | P-mmol/gVSS.h | 0.05 (R^2^=0.97; F1,2=144;P<0.001) | NA | 0.05 (R^2^=0.92; F1,2=42;P<0.010) |
|  | $q_{PO4,Ox}$ | P-mmol/gVSS.h | 1.06 (R^2^=0.96; F1,5=273;P<0.001) | 0.25 (R^2^=0.98; F1,5=215;P<0.001) | 0.21 (R^2^=0.97; F1,5=88;P<0.001) |

- ANOVA test run with a 95 % confidence interval

## Calculation of rates, stoichiometry and statistics

The stoichiometric ratios were calculated based on the observed net conversions between their initial and final concentrations during the anaerobic phase. The kinetics rates were calculated via linear regression (Figure S2) as described in Smolders et al. (1995). In order to assess the confidence interval the ANOVA test was used as shown below. The observed growth rate and mass balances were calculated as described in Henze et al. (2008) based in all corresponding inflows and outflows during the cycle analysis.

## Figure S2

Figure S2.- Example of statistical analysis calculation of the transformation rates

| \|  \| \| --- \| |  |  |  |  |  |  |  |  |
| --- | --- | --- | --- | --- | --- | --- | --- | --- | --- |
|  |  |  |  |  |  |  |  |  |
|  |  |  |  |  |  |  |  |  |
|  |  |  |  |  |  |  |  |  |
|  |  |  |  |  |  |  |  |  |
|  |  |  |  |  |  |  |  |  |
|  |  |  |  |  |  |  |  |  |
|  |  |  |  |  |  |  |  |  |
|  |  |  |  |  |  |  |  |  |
|  |  |  |  |  |  |  |  |  |
|  |  |  |  |  |  |  |  |  |
|  |  |  |  |  |  |  |  |  |
|  |  |  |  |  |  |  |  |  |
|  |  |  |  |  |  |  |  |  |
|  |  |  |  |  |  |  |  |  |
|  |  |  |  |  |  |  |  |  |
|  |  |  |  |  |  |  |  |  |
| SUMMARY OUTPUT | |  |  |  |  |  |  |  |
|  |  |  |  |  |  |  |  |  |
|  |  |  |  |  |  |  |  |  |
| *Regression Statistics* | |  |  |  |  |  |  |  |
| Multiple R | 0.998426 |  |  |  |  |  |  |  |
| R Square | 0.996854 |  |  |  |  |  |  |  |
| Adjusted R Square | 0.996068 |  |  |  |  |  |  |  |
| Standard Error | 0.040235 |  |  |  |  |  |  |  |
| Observations | 6 |  |  |  |  |  |  |  |
|  |  |  |  |  |  |  |  |  |
| ANOVA |  |  |  |  |  |  |  |  |
|  | *df* | *SS* | *MS* | *F* | *Significance F* |  |  |  |
| Regression | 1 | 2.051907 | 2.051907 | 1267.485 | 3.72E-06 |  |  |  |
| Residual | 4 | 0.006476 | 0.001619 |  |  |  |  |  |
| Total | 5 | 2.058383 |  |  |  |  |  |  |
|  |  |  |  |  |  |  |  |  |
|  | *Coefficients* | *Standard Error* | *t Stat* | *P-value* | *Lower 95%* | *Upper 95%* | *Lower 95.0%* | *Upper 95.0%* |
| Intercept | 2.267626 | 0.030288 | 74.86868 | 1.91E-07 | 2.183533 | 2.351719 | 2.183533 | 2.351719 |
| X Variable 1 | -2.71788 | 0.076341 | -35.6018 | 3.72E-06 | -2.92984 | -2.50592 | -2.92984 | -2.50592 |
